# Quantum channel measurement with local quantum Bernoulli noises

**DOI:** 10.1038/s41598-022-17268-y

**Published:** 2022-07-28

**Authors:** Qi Han, Yanan Han, Yaxin Kou, Ning Bai

**Affiliations:** College of Mathematics and Statistics, Northwest Normal University, Lanzhou, 730070 Gansu China

**Keywords:** Quantum information, Applied mathematics

## Abstract

As an important stochastic process, quantum Bernoulli noises has a very important physical background and is an important research object in the field of quantum information. In this paper, we review local quantum Bernoulli noises and local quantum mutual entropy, then introduce quantum channel measurement with local quantum Bernoulli noises. On this basis, we give the channel structure between two systems, and prove the completely positivity of this quantum channel. We also give a channel application on the local quantum mutual entropy.

## Introduction

Quantum information is a hot topic in current research. As an important tool of accurate measurement in the field of quantum information, quantum channel has also received broad attentions. The channel is one of the important communication theory bases because it has an essential physical meaning, that is, when a person wants to send a message, it must through a certain channel, and the mutual entropy discussed earlier also depends on the channel. In a closed quantum system, the transmission of information follows *U*-evolution. But in general, for an open quantum system, the transmission of information will be interfered by noise. In this paper, we consider the problem of information transmission under the influence of local quantum Bernoulli noise. A general quantum system is described by a $C^{*}$-algebra or a Von Neumann algebra, here we discuss the channel transformation in $C^{*}$-algebra contexts.

Privault^1^ introduced stochastic analysis of Bernoulli processes and its applications. Later, Wang, Chai, and Lu^2^ introduces quantum Bernoulli noises (QBNs) in discrete time which are the family of local annihilation and local creation operator acting on Bernoulli functionals. Wang and Zhang^3^ constructed Dirichlet forms from annihilation operators on Bernoulli functionals, and introduced a new type of QBNs which called localization of QBNs (short as LQBNs). Let’s briefly review Han, Chen and Lu^4^ introduced to local quantum entropy $S(\rho _{k})$ of quantum Bernoulli noises. In this paper, on the basis of Han, Han, Kou and Lu^5^, the mathematical structure of the quantum channel is studied when the quantum Bernoulli noise is localized.

Quantum channel refers to the part through which information travels from the input system to the output system. There is noise in the channel, even if the input signal is zero, the output signal still has a certain power. Quantum channels differ from classical channels in that they use qubit. A qubit is a quantum object in a superposition state, kind of like a superposition of 1 and 0, that is, before measurement, it can be any mixture of 1 and 0, so the possible value is infinite, that is the key of the quantum channel. When we use local quantum Bernoulli noise, we make a measurement of the information, and the qubit are no longer superimposed after the measurement, so the measurement result is classical.

Let $(\Omega ,{\mathcal {F}},{\mathbb {P}})$ be a probability space, $L^{2}(\Omega ,{\mathcal {F}},{\mathbb {P}})$ the usual Hilbert space of square integrable complex-valued functions on $(\Omega ,{\mathcal {F}},{\mathbb {P}})$. Let ${\mathcal {A}}=B({\mathcal {H}})$ be the set of all bounded operators on a separable Hilbert space ${\mathcal {H}}$. ${\mathcal {G}}={\mathcal {G}}({\mathcal {H}})$ is the set of all canonical states (density operators) on ${\mathcal {A}}$. In order to discuss the communication process, we need two dynamical systems: An input system $({\mathcal {A}}_{1},{\mathcal {G}}_{1})$ and an output system $({\mathcal {A}}_{2},{\mathcal {G}}_{2})$ acting on the Hilbert spaces ${\mathcal {H}}_{1}$ and ${\mathcal {H}}_{2}$, respectively.

The arrangement of this article is as follows. In Sect. “Preliminary knowledge”, we briefly recall the basic concepts and properties of QBNs and LQBNs. In Sect. “Channel construction with local quantum Bernoulli noises”, We give the mathematical structure of the communication channel when the noise is quantum Bernoulli noises, and prove that the channel is completely positive. In Sect. “Channel measurement of local quantum mutual entropy”, we make a simple measurement of this channel. In Sect. “Summary”, a brief summary.

## Preliminary knowledge

In this section, we firstly recall main concepts and properties about QBNs, which play an important role in our following discussion. We refer to Wang, Chai and Lu^1^ Han, Han and Kou^5^ for details.

Symbols description: Let ${\mathbb {N}}$ be the set of all non-negative integers, $\Gamma$ the finite power set of ${\mathbb {N}}$, namely,

2.1 $$\begin{aligned} \Gamma =\{\sigma \mid \sigma \subset {\mathbb {N}} ~and ~\sharp (\sigma )<\infty \}, \end{aligned}$$

where $\sharp (\sigma )$ denotes the cardinality of $\sigma$ as a set. In this paper, both *j* and *k* represent non-negative integers belonging to ${\mathbb {N}}$. If ${\mathcal {H}}$ be a Hilbert space, then ${{\textbf {B}}}({\mathcal {H}})$ represents the set of all bounded operators, and ${\mathcal {G}}={\mathcal {G}}({\mathcal {H}})$ is the set of all canonical states (density operators) on ${{\textbf {B}}}({\mathcal {H}})$. We denote by $\langle \cdot ,\cdot \rangle$ the usual inner product of the space ${\mathcal {H}}$ and by $\parallel \cdot \parallel$ the corresponding norm.

In reference Wang^1^, ${\mathfrak {h}}$ be the space of square integrable complex-valued Bernoulli functionals, namely ${\mathfrak {h}}=L^{2}(\Omega ,{\mathcal {F}}, {\mathbb {P}})$, thus ${\mathfrak {h}}$ has $\{Z_{\sigma }\mid \sigma \in \Gamma \}$ as its orthonormal basis, where $Z_{\emptyset }=1$ and

2.2 $$\begin{aligned} Z_{\sigma }=\prod _{j\in \sigma }Z_{j},\sigma \in \Gamma ,\sigma \ne \emptyset . \end{aligned}$$

Therefore, we introduce the following lemma and definitions.

### Lemma 2.1

^1^ For $k\in {\mathbb {N}}$, there is a bounded linear operator $\partial _{k}$ on ${\mathfrak {h}}$ , such that

2.3 $$\begin{aligned} \partial _{k}Z_{\sigma }= & {} {\mathbf {1}}_{\sigma }(k)Z_{\sigma \setminus k},~\sigma \in \Gamma , \end{aligned}$$

2.4 $$\begin{aligned} \partial _{k}^{*}Z_{\sigma }= & {} [1-{\mathbf {1}}_{\sigma }(k)]Z_{\sigma \cup k},~ \sigma \in \Gamma , \end{aligned}$$

where $\sigma \setminus k=\sigma \setminus \{k\}, \sigma \cup k=\sigma \cup \{k\}$, ${\mathbf {1}}_{\sigma }(k)$ the indicator of $\sigma$ as a subset of ${\mathbb {N}}$ and $\partial _{k}^{*}$ is the adjoint of operator $\partial _{k}$.

The operator $\partial _{k}$ and its adjoint $\partial _{k}^{*}$ are usually known as the annihilation and creation operators acting on Bernoulli functionals, respectively.

### Definition 2.2

^1^ The family $\{{\partial _{k}, \partial _{k}^{*}}\}_{k\ge 0}$ of annihilation and creation operators is called quantum Bernoulli noises.

Let $\rho _{k}$ be the density operator on ${\mathfrak {h}}=L^{2}(\Omega ,{\mathcal {F}}, {\mathbb {P}})=L^{2}(\Omega )$, and the orthonormal basis of ${\mathfrak {h}}$ be $\{Z_{\sigma }\mid \sigma \in \Gamma \}$, then the expression of $\rho _{k}$ is

2.5 $$\begin{aligned} \rho _{k}=\sum _{k=1}^{{\mathbb {N}}}\lambda _{k}|Z_{\sigma _{k}}\rangle \langle Z_{\sigma _{k}}|, \end{aligned}$$

where $\sum _{k}\lambda _{k}=1$, and $|Z_{\sigma _{k}}\rangle \langle Z_{\sigma _{k}}|=E_{k}$ is the projection operator.

### Definition 2.3

^6^ For $\rho$ is a density operator, its quantum entropy is defined as

2.6 $$\begin{aligned} S(\rho )\equiv -Tr(\rho \log \rho ), \end{aligned}$$

and its quantum relative entropy

2.7 $$\begin{aligned} S(\rho \parallel \sigma ) \equiv Tr\rho (\log \rho -\log \sigma ), \end{aligned}$$

where the logarithms indicated by $\log$ are taken to base two. If $\lambda _x$ are the eigenvalues of $\rho$ then Von Neumann’s definition can be re-expressed

2.8 $$\begin{aligned} S(\rho )=-\sum \limits _{x}\lambda _x\log \lambda _x, \end{aligned}$$

where we define $0\log 0\equiv 0$, as for the Shannon entropy.

A channel from the input system to the output system is a mapping $\Lambda ^{*}$ from ${\mathcal {G}}({\mathfrak {h}})~\rightarrow ~{\mathcal {G}}({\mathfrak {h}})$. An input state $\rho \in {\mathcal {G}}({\mathfrak {h}})$ is sent to the output system through a channel $\Lambda ^{*}$, so that the output state is written as ${\widetilde{\rho }}\equiv \Lambda ^{*}\rho$. So we introduce the following definition.

### Definition 2.4

^5^ The compound state $\Phi _{kE}$ (corresponding to a joint state in classical systems) of $\rho _{k}$ and $\Lambda ^{*}\rho _{k}$ on the space $L^{2}(\Omega )$ was given by

2.9 $$\begin{aligned} \Phi _{kE}=\sum _{i}\nu _{i}E_{i}\otimes \Lambda ^{*}E_{i}, \end{aligned}$$

where *E* stands for a Schatten decomposition $\{E_{i}\}$ of $\rho _{k}$ and $\nu _{i}$ was a eigenvalue of $\rho _{k}$.

Applying the relative entropy $S(\cdot \Vert \cdot )$ and two compound states $\Phi _{kE}$, $\Phi _{k0}\equiv \rho _{k}\otimes \Lambda ^{*}\rho _{k}$ (the former includes a certain correlation of input and output and the later does not), so we have the following lemma.

### Lemma 2.5

^5^ If max $\sigma \le k,~\sigma \in \Gamma ,~k\in {\mathbb {N}}$, where max $\sigma$ stand for the greatest element in $\sigma$. $\rho _{k}$ is a density operator on $L^{2}(\Omega )$. Then the quantum mutual entropy in terms of local quantum Bernoulli noises is

2.10 $$\begin{aligned} I(\rho _{k};\Lambda ^{*})=sup\{\sum _{i}\nu _{i}S(\Lambda ^{*}E_{i}\Vert \Lambda ^{*}\rho _{k}); E=\{E_{i}\}\}, \end{aligned}$$

where the supremum is taken over all Schatten decompositions of $\rho _{k}$ because this decomposition is not always unique unless every eigenvalue of $\rho _{k}$ is not degenerated.

## Channel construction with local quantum Bernoulli noises

In this section we propose the mathematical structure of channels that describe some of the communication processes.

Let ${\mathcal {A}}={{\textbf {B}}}({\mathcal {H}})$ be the set of all bounded operators on a separable Hilbert space ${\mathcal {H}}=L^{2}(\Omega ,{\mathcal {F}}, {\mathbb {P}})$, that is ${\mathcal {A}}$ be a input $C^{*}$-algebra and ${\mathcal {G}}({\mathcal {A}})$ be the set of all states on ${\mathcal {A}}$. If $\Lambda :{\mathcal {A}}_{2}\rightarrow \mathcal {{\mathcal {A}}}$ is a map from an algebra ${\mathcal {A}}_{2}$ to an algebra ${\mathcal {A}}$ where ${\mathcal {A}}_{2}$ output $C^{*}$-algebra, then its dual map $\Lambda ^{*}:{\mathcal {G}}({\mathcal {A}})~\rightarrow ~{\mathcal {G}}({\mathcal {A}}_{2})$ is called a channel.

In order to discuss the communication process, we need two dynamical systems: An input system $({\mathcal {A}}_{1},{\mathcal {G}}_{1})$ and an output system $({\mathcal {A}}_{2},{\mathcal {G}}_{2})$ acting on the Hilbert space ${\mathcal {H}}_{1}$ and ${\mathcal {H}}_{2}$, respectively. Later in paper, we consider the input space and the output space to be the same space, that is, ${\mathcal {H}}_{1}={\mathcal {H}}_{2}={\mathcal {H}}$. Here, we consider the direct effects of local quantum Bernoulli noises and loss to determine the general form of the channel in the communication process, channels exist during propagation, in addition to the Hilbert space ${\mathcal {H}}$, we here use two more Hilbert spaces ${\mathfrak {h}}$ and ${\mathcal {K}}$ in order to describe explicitly some external effects to the input and output states. For instance a state in ${\mathfrak {h}}$ induces local quantum Bernoulli noises into the channel and a state in ${\mathcal {K}}$ indicates a loss of information at the output system.

Let $\xi \in {\mathcal {G}}({\mathfrak {h}})$ be a state describing the LQBNs in the channel. We consider the following maps:

3.1 $$\begin{aligned} {{\textbf {B}}}({\mathcal {H}}){\mathop {\longrightarrow }\limits ^{a}} {{\textbf {B}}}({\mathcal {H}}\otimes {\mathcal {K}}){\mathop {\longrightarrow }\limits ^{\pi }} {{\textbf {B}}}({\mathcal {H}}\otimes {\mathfrak {h}}){\mathop {\longrightarrow }\limits ^{\gamma }} {{\textbf {B}}}({\mathcal {H}}). \end{aligned}$$

These maps $a, \pi , \gamma$ are defined as follows.

1. The map *a* is an amplification from ${{\textbf {B}}}({\mathcal {H}})$ to ${{\textbf {B}}}({\mathcal {H}}\otimes {\mathcal {K}})$ given by $a(A)=A\otimes I$ for any $A\in {{\textbf {B}}}({\mathcal {H}})$, $I\in {{\textbf {B}}}({\mathcal {K}})$.
2. The map $\pi$ is a completely positive map from ${{\textbf {B}}}({\mathcal {H}}\otimes {\mathcal {K}})$ to ${{\textbf {B}}}({\mathcal {H}}\otimes {\mathfrak {h}})$, $\pi (I)=1$ describes the physical mechanism of the channel, $I\in {{\textbf {B}}}({\mathcal {H}}\otimes {\mathcal {K}})$.
3. The map $\gamma$ is from ${{\textbf {B}}}({\mathcal {H}}\otimes {\mathfrak {h}})$ to ${{\textbf {B}}}({\mathcal {H}})$ given by $\gamma (Q)=Tr_{{\mathfrak {h}}}\xi Q$ for any $Q\in {{\textbf {B}}}({\mathcal {H}}\otimes {\mathfrak {h}})$, where $Tr_{{\mathfrak {h}}}$ is a partial trace with respect to the Hilbert space ${\mathfrak {h}}$, where $\xi$ be a localization of quantum Bernoulli noises(LQBNs).

Then we define the mapping $\Lambda$ from ${{\textbf {B}}}({\mathcal {H}})$ to ${{\textbf {B}}}({\mathcal {H}})$ such that

3.2 $$\begin{aligned} \Lambda =\gamma \circ \pi \circ a. \end{aligned}$$

It is easy to show Ohya^7^that these maps are completely positive, that is, the mappings $\gamma$, $\pi$ and *a* are completely positive, hence $\Lambda =\gamma \circ \pi \circ a$ is also completely positive.

We next consider the dual maps of *a*, $\pi$ and $\gamma$.

- The dual map $a^{*}$ of *a* is a map from ${\mathcal {G}}({\mathcal {H}}\otimes {\mathcal {K}})$ to ${\mathcal {G}}({\mathcal {H}})$ such that $a^{*}(\theta )=Tr_{{\mathcal {K}}}\theta$ for any $\theta \in {\mathcal {G}}({\mathcal {H}}\otimes {\mathcal {K}})$.
- The dual map $\pi ^{*}$: ${\mathcal {G}}({\mathcal {H}}\otimes {\mathfrak {h}})\rightarrow {\mathcal {G}}({\mathcal {H}}\otimes {\mathcal {K}})$ is given by $Tr\pi ^{*}(\sigma )W=Tr\sigma \pi (W)$ for any $\sigma \in {\mathcal {G}}({\mathcal {H}}\otimes {\mathfrak {h}})$ and any $W\in {{\textbf {B}}}({\mathcal {H}}\otimes {\mathcal {K}})$.
- The dual map $\gamma ^{*}$: ${\mathcal {G}}({\mathcal {H}})\rightarrow {\mathcal {G}}({\mathcal {H}}\otimes {\mathfrak {h}})$ is given by $Tr\gamma ^{*}(\rho _{k})Q=Tr\rho _{k}\gamma (Q)$ for any $\rho _{k}\in {\mathcal {G}}({\mathcal {H}})$ and any $Q\in {{\textbf {B}}}({\mathcal {H}}\otimes {\mathfrak {h}})$, where $\rho _{k}$ is a input state.

It is easily seen that $\gamma ^{*}$ is expressed as $\gamma ^{*}(\rho _{k})=\rho _{k}\otimes \xi$, where $\xi$ be a localization of quantum Bernoulli noises(LQBNs).

Therefore, once we know the LQBNs $\xi$ and the mechanism of the transformation $\pi$, we can write down a channel explicitly such that

3.3 $$\begin{aligned} \Lambda ^{*}=a^{*}\circ \pi ^{*}\circ \gamma ^{*} \end{aligned}$$

or equivalently

3.4 $$\begin{aligned} \Lambda ^{*}\rho _{k}=Tr_{{\mathcal {K}}}\pi ^{*}(\rho _{k}\otimes \xi ), \end{aligned}$$

for any $$\rho _{k}\in {\mathcal {G}}({\mathfrak {h}})$$.

We now build a more specific channel model for quantum Bernoulli noises processes. A quantum system composed of photons is described by the Hamiltonian $H=b^{*}b+\frac{1}{2}$, where $b^{*}$ and *b* are creation and annihilation operators of a photon, respectively. Here we borrow the Schrodinger equation mentioned by Ohya^8^: $Hx(q)=Ex(q)$, the eigenvalue $E_{n}=n+\frac{1}{2}(n\ge 0)$ and the eigenvector $x_{n}(q)=(1/(\pi ^{1/2}n!)^{1/2})H_{n}\times (2^{1/2}q)exp(-q^{2}/2)$, where $H_{n}(q)$ is the *n*th Hermite function. Our photon communication process can be considered as follows: when $n_{1}$ photons are transmitted from the input system, $m_{1}$ photons from the noise system add to the signal. Then $m_{2}$ photons are lost to the loss system through the channel, and $n_{2}$ photons are detected in the output system. Simply write down the coordinates of the spaces ${\mathcal {H}}_{1}={\mathcal {H}}_{2}={\mathcal {H}}$, ${\mathfrak {h}}$, ${\mathcal {K}}$ in this model. $\{|Z_{\sigma }^{(1)}\rangle \}$ and *q* are the completely orthonormal system(CONS) and coordinate of ${\mathcal {H}}$, respectively; $\{|Z_{\sigma }\rangle \}$ and *t* are the CONS and coordinate of ${\mathfrak {h}}$, respectively. Similarly, we have $\{|Z_{\sigma }^{(2)}\rangle \}$ and *s*.

For simplicity, we put $m_{1}=0$. Let the local quantum Bernoulli noise $\xi =|Z_{\sigma }\rangle \langle Z_{\sigma }|\in {\mathcal {G}}({\mathfrak {h}})$, where $|Z_{\sigma }\rangle$ is some vector in the local quantum Bernoulli noise system. We define the mapping $\pi ^{*}(\bullet )=U_{t}(\bullet )U_{t}^{*}$, where $t\in R,~U_{t}=exp(-itH)$, *H* is the Hamiltonian of the system.

Therefore, the channel can be represented as

3.5 $$\begin{aligned} \Lambda ^{*}\rho _{k}&=a^{*}\circ \pi ^{*}\circ \gamma ^{*}\rho _{k}\\&=Tr_{{\mathcal {K}}}\pi ^{*}(\rho _{k}\otimes \xi )\\&=Tr_{{\mathcal {K}}}U_{t}(\rho _{k}\otimes \xi )U_{t}^{*}. \end{aligned}$$

We want to prove that $\Lambda ^{*}$ is a completely positive mapping, we have only to show the completely positivity of $\gamma ^{*}$ and $a^{*}$ because $\pi ^{*}$ is completely positive.

Before we can prove the positivity of the channel, we need the following lemma.

### Lemma 3.1

^7^ Let $({\mathcal {A}},{\mathcal {G}}({\mathcal {A}}))$ be an input system and $(\overline{{\mathcal {A}}},\overline{{\mathcal {G}}({\mathcal {A}})})$ be an output system. Take any $\varphi , \psi ~\in {\mathcal {G}}({\mathcal {A}})$.

1, $\Lambda ^{*}$ is linear if $\Lambda ^{*}(\lambda \varphi +(1-\lambda )\psi )=\lambda \Lambda ^{*}\varphi +(1-\lambda )\Lambda ^{*}\psi$ holds for any $\lambda \in [0,1]$.

2, $\Lambda ^{*}$ is completely positive (CP) if $\Lambda ^{*}$ is linear and its dual $\Lambda : \overline{{\mathcal {A}}}\rightarrow {\mathcal {A}}$ satisfies

3.6 $$\begin{aligned} \sum _{i,j=1}^{n}A_{i}^{*}\Lambda (\overline{A_{i}^{*}}\overline{A_{j}})A_{j}\ge 0 \end{aligned}$$

for any $n\in {\mathbb {N}}$ and any $\{\overline{A_{i}}\}\subset \overline{{\mathcal {A}}}$, $\{A_{i}\}\subset {\mathcal {A}}$.

### Proposition 3.2

The mapping $\gamma ^{*}:\rho _{k}\rightarrow \rho _{k}\otimes \xi$ have a completely positivity and trace-preserving property.

### Proof

For any $A_{i}\in B({\mathcal {H}}\otimes {\mathfrak {h}})$, $B_{j}\in B({\mathcal {H}})$, where $i,~j\in {\mathbb {N}}$, $\{|Z_{\sigma _{k}}^{(1)}\rangle \}$ and $\{|Z_{\sigma _{l}}\rangle \}$ are the CONS of ${\mathcal {H}}$ and ${\mathfrak {h}}$, respectively. $\xi \in {\mathcal {G}}({\mathfrak {h}})$, $Z_{\sigma }^{(1)}\in {\mathcal {H}}$, any $n\in {\mathbb {N}}$, we have

$$\begin{aligned} \left\langle {Z_{\sigma }^{{(1)}} ,\sum\limits_{{i,j = 1}}^{n} {B_{i}^{*} } \gamma (A_{i}^{*} A_{j} )B_{j} Z_{\sigma }^{{(1)}} } \right\rangle & = \sum\limits_{{i,j = 1}}^{n} {\left\langle {B_{i} Z_{\sigma }^{{(1)}} ,tr_{\mathrm{\mathcal{K}}} \xi A_{i}^{*} A_{j} B_{j} Z_{\sigma }^{{(1)}} } \right\rangle } \\ & = \sum\limits_{{i,j = 1}}^{n} {\sum\limits_{m} {\left\langle {B_{i} Z_{\sigma }^{{(1)}} \otimes Z_{{\sigma _{m} }} ,(I \otimes \xi )A_{i}^{*} A_{j} B_{j} Z_{\sigma }^{{(1)}} \otimes Z_{{\sigma _{m} }} } \right\rangle } } \\ & = \sum\limits_{{k,l}} {\sum\limits_{{i,j = 1}}^{n} {\sum\limits_{m} {\left\langle {B_{i} Z_{\sigma }^{{(1)}} \otimes Z_{{\sigma _{m} }} ,(I \otimes \xi )A_{i}^{*} |Z_{{\sigma _{k} }}^{{(1)}} \otimes Z_{{\sigma _{l} }} } \right\rangle \left\langle {Z_{{\sigma _{k} }}^{{(1)}} \otimes Z_{{\sigma _{l} }} |A_{j} B_{j} Z_{\sigma }^{{(1)}} \otimes Z_{{\sigma _{m} }} } \right\rangle } } } \\ & = \sum\limits_{{i,j = 1}}^{n} {\sum\limits_{{k,l}} {\left\langle {Z_{{\sigma _{k} }}^{{(1)}} \otimes Z_{{\sigma _{l} }} ,(B_{j} Z_{\sigma }^{{(1)}} B_{i} Z_{\sigma }^{{(1)}} \otimes I)(I \otimes \xi )A_{i}^{*} Z_{{\sigma _{k} }}^{{(1)}} \otimes Z_{{\sigma _{l} }} } \right\rangle } } \\ & = \sum\limits_{{k,l}} {\sum\limits_{{i,j = 1}}^{n} {\left\langle {Z_{{\sigma _{k} }}^{{(1)}} \otimes Z_{{\sigma _{l} }} ,A_{j} (B_{j} \otimes I)(|Z_{\sigma }^{{(1)}} } \right\rangle } } \left\langle {Z_{\sigma }^{{(1)}} | \otimes I)(B_{i}^{*} \otimes I)(I \otimes \xi ^{{\frac{1}{2}}} )(I \otimes \xi ^{{\frac{1}{2}}} ) \times A_{i}^{*} Z_{{\sigma _{k} }}^{{(1)}} \otimes Z_{{\sigma _{l} }} } \right\rangle \\ & = \sum\limits_{{k,l}} {\sum\limits_{{j = 1}}^{n} {\left\langle {Z_{{\sigma _{k} }}^{{(1)}} \otimes Z_{{\sigma _{l} }} ,A_{j} (I \otimes \xi ^{{\frac{1}{2}}} )(B_{j} \otimes I)Z_{\sigma }^{{(1)}} \otimes Z_{{\sigma _{l} }} } \right\rangle } } \overline{{ \times \sum\limits_{{i = 1}}^{n} {\left\langle {Z_{{\sigma _{k} }}^{{(1)}} \otimes Z_{{\sigma _{l} }} ,A_{i} (I \otimes \xi ^{{\frac{1}{2}}} )(B_{i} \otimes I)Z_{\sigma }^{{(1)}} \otimes Z_{{\sigma _{l} }} } \right\rangle } }} \\ & = \sum\limits_{{k,l}} {\left| {\sum\limits_{{j = 1}}^{n} {\left\langle {Z_{{\sigma _{k} }}^{{(1)}} \otimes Z_{{\sigma _{l} }} ,A_{j} (I \otimes \xi ^{{\frac{1}{2}}} )(B_{j} \otimes I)Z_{\sigma }^{{(1)}} \otimes Z_{{\sigma _{l} }} } \right\rangle } } \right|} ^{2} \\ & \ge 0. \\ \end{aligned}$$

According to lemma 3.1 and $Tr(\sum _{i,j=1}^{n}B_{i}^{*}\gamma (A_{i}^{*}A_{j})B_{j})=\langle Z_{\sigma }^{(1)},\sum _{i,j=1}^{n}B_{i}^{*}\gamma (A_{i}^{*}A_{j})B_{j}Z_{\sigma }^{(1)}\rangle$, therefore, we have that $\gamma ^{*}$ is a completely positivity and trace-preserving property map. $\square$

### Proposition 3.3

The mapping $a^{*}:\theta \rightarrow Tr_{{\mathcal {K}}}\theta$ have a completely positivity and trace-preserving property.

### Proof

For any $A_{i}\in B({\mathcal {H}})$, $B_{j}\in B({\mathcal {H}}\otimes {\mathcal {K}})$, where $i,~j\in {\mathbb {N}}$, $\{|Z_{\sigma _{k}}^{(1)}\rangle \}$ and $\{|Z_{\sigma _{l}}^{(2)}\rangle \}$ are the CONS of ${\mathcal {H}}$ and ${\mathcal {K}}$, respectively. $Z_{\sigma }^{(1)}\otimes Z_{\sigma }^{(2)}\in {\mathcal {H}}\otimes {\mathcal {K}}$, for any $n\in {\mathbb {N}}$, we have

$$\begin{aligned} \left\langle {Z_{\sigma }^{{(1)}} \otimes Z_{\sigma }^{{(2)}} ,\sum\limits_{{i,j = 1}}^{n} {B_{i}^{*} } a(A_{i}^{*} A_{j} )B_{j} Z_{\sigma }^{{(1)}} \otimes Z_{\sigma }^{{(2)}} } \right\rangle & = \sum\limits_{{i,j = 1}}^{n} {\left\langle {B_{i} Z_{\sigma }^{{(1)}} \otimes Z_{\sigma }^{{(2)}} ,(A_{i}^{*} A_{j} \otimes I)B_{j} Z_{\sigma }^{{(1)}} \otimes Z_{\sigma }^{{(2)}} } \right\rangle } \\ & = \sum\limits_{{i,j = 1}}^{n} {\left\langle {B_{i} Z_{\sigma }^{{(1)}} \otimes Z_{\sigma }^{{(2)}} ,(A_{i}^{*} \otimes I)(A_{j} \otimes I)B_{j} Z_{\sigma }^{{(1)}} \otimes Z_{\sigma }^{{(2)}} } \right\rangle } \\ & = \left\langle {\sum\limits_{{i = 1}}^{n} {(A_{i} \otimes I)} B_{i} Z_{\sigma }^{{(1)}} \otimes Z_{\sigma }^{{(2)}} ,\sum\limits_{{j = 1}}^{n} {(A_{j} \otimes I)} B_{j} Z_{\sigma }^{{(1)}} \otimes Z_{\sigma }^{{(2)}} } \right\rangle \\ & = \sum\limits_{{k,l}} {\left\langle {\sum\limits_{{i = 1}}^{n} {(A_{i} \otimes I)} B_{i} Z_{\sigma }^{{(1)}} \otimes Z_{\sigma }^{{(2)}} ,\sum\limits_{{j = 1}}^{n} {(A_{j} \otimes I)} B_{j} Z_{\sigma }^{{(1)}} \otimes Z_{\sigma }^{{(2)}} } \right\rangle } \left| {Z_{{\sigma _{k} }}^{{(1)}} \otimes Z_{{\sigma _{l} }}^{{(2)}} \langle Z_{{\sigma _{k} }}^{{(1)}} \otimes Z_{{\sigma _{l} }}^{{(2)}} } \right| \\ & = \sum\limits_{{k,l}} {\left\langle {\sum\limits_{{i = 1}}^{n} {(A_{i} \otimes I)} B_{i} Z_{\sigma }^{{(1)}} \otimes Z_{\sigma }^{{(2)}} ,Z_{{\sigma _{k} }}^{{(1)}} \otimes Z_{{\sigma _{l} }}^{{(2)}} } \right\rangle \times \left\langle {Z_{{\sigma _{k} }}^{{(1)}} \otimes Z_{{\sigma _{l} }}^{{(2)}} ,\sum\limits_{{j = 1}}^{n} {(A_{j} \otimes I)} B_{j} Z_{\sigma }^{{(1)}} \otimes Z_{\sigma }^{{(2)}} } \right\rangle } \\ & = \sum\limits_{{k,l}} {\left\langle {\sum\limits_{{i = 1}}^{n} {(A_{i} \otimes I)} B_{i} Z_{\sigma }^{{(1)}} \otimes Z_{\sigma }^{{(2)}} ,Z_{{\sigma _{k} }}^{{(1)}} \otimes Z_{{\sigma _{l} }}^{{(2)}} } \right\rangle } \times \overline{{\left\langle {\sum\limits_{{j = 1}}^{n} {(A_{j} \otimes I)} B_{j} Z_{\sigma }^{{(1)}} \otimes Z_{\sigma }^{{(2)}} ,Z_{{\sigma _{k} }}^{{(1)}} \otimes Z_{{\sigma _{l} }}^{{(2)}} } \right\rangle }} \\ & = \sum\limits_{{k,l}} {\left| {\left\langle {\sum\limits_{{i = 1}}^{n} {(A_{i} \otimes I)} B_{i} Z_{\sigma }^{{(1)}} \otimes Z_{\sigma }^{{(2)}} ,Z_{{\sigma _{k} }}^{{(1)}} \otimes Z_{{\sigma _{l} }}^{{(2)}} \rangle } \right\rangle } \right|} ^{2} \\ & \ge 0. \\ \end{aligned}$$

For the same reason as proposition 3.2, we have that $a^{*}$ is a completely positivity and trace-preserving property map. $\square$

Since the composition of completely positive maps is completely positive, from propositions 3.2 and 3.3, we have the following corollary similar to Choi-Kraus theorem^9^.

### Corollary 3.4

The mapping $\Lambda$ is completely positivity and trace-preserving, therefore, $\Lambda ^{*}$ is a channel.

Of course, in addition to the complete positivity and trace-preserving in this section, channels also have other properties, such as ergodicity, disorder, determinism and so on. These properties, which we will discuss in a later article, are not explained here.

## Channel measurement of local quantum mutual entropy

In this section, we have made a simple channel measurement, that is, the amount of correct information transmitted through the channel when the noise is quantum Bernoulli noise, which we here call local quantum mutual entropy.

Because of conservation of energy (photon number), then a relation $n_{1}+n_{2}=m_{1}+m_{2}$ should hold. According to Ohya^7^, we also apply following linear transformation among the coordinates *q*, *t*, *s* of the input; noise; output and loss systems, respectively:

4.1 $$\begin{aligned} q=\frac{\beta t}{1-\alpha },~~s=-\beta q+\alpha t,~~~\alpha \ne 1 \end{aligned}$$

where $$\alpha ^{2}+\beta ^{2}=1$$

For simplicity, we put $m_{1}=0$. By using this linear transformation and we define the mapping $\pi ^{*}\bullet =U_{t}(\bullet )U_{t}^{*}$, hence the local quantum noise source is described by a state $\xi =|Z_{\sigma }\rangle \langle Z_{\sigma }|\in {\mathcal {G}}({\mathfrak {h}})$, where $|Z_{\sigma }\rangle$ is some vector in the local quantum Bernoulli noise system for an input state $E_{n}=|Z_{\sigma _{n}}^{(1)}\rangle \langle Z_{\sigma _{n}}^{(1)}|$ such that

4.2 $$\begin{aligned} U_{t}(Z_{\sigma }^{(1)}\otimes Z_{\sigma }^{(1)})(q,s)&=Z_{\sigma }^{(1)}\otimes Z_{\sigma }^{(1)}(\alpha q-\beta s,\beta q+\alpha s)~~(=\Psi _{n}(Z_{\sigma _{1}},Z_{\sigma _{2}}))\\&=\sum _{j=0}^{n}c_{j}^{n}Z_{\sigma _{j}}^{(1)}\otimes Z_{\sigma _{n-j}}^{(2)}(q,s), \end{aligned}$$

where $c_{j}^{n}=\sqrt{n!/j!(n-j)!}(-\beta )^{n-j}\alpha ^{j}$, we have

4.3 $$\begin{aligned} \Lambda ^{*}E_{n}=tr_{{\mathcal {K}}}U_{t}(E_{n\otimes \xi })U_{t}^{*}=tr_{{\mathcal {K}}}|\Psi _{n}\rangle \langle \Psi _{n}|= \sum _{j=0}^{n}|c_{j}^{n}|^{2}|Z_{\sigma _{j}}^{(2)}\rangle \langle Z_{\sigma _{j}}^{(2)}|. \end{aligned}$$

Therefore for an input state $\rho _{k}=\sum _{m=0}^{N}\lambda _{m}E_{m}(0\le N\le \infty )$ with $\sum _{m}\lambda _{m}=1$ and $\lambda _{p}\ne \lambda _{j} (p\ne j)$, where $E_{m}=|Z_{\sigma _{m}}^{(1)}\rangle \langle Z_{\sigma _{m}}^{(1)}|$. The compound state and the trivial compound state introduced in preliminary knowledge are given by

4.4 $$\begin{aligned} \Phi _{kE}= & {} \sum _{m}\lambda _{m}E_{m}\otimes \Lambda ^{*}E_{m}=\sum _{m}\lambda _{m}E_{m}\otimes \Lambda ^{*}E_{m}= \sum _{m=0}^{N}\sum _{j=0}^{m}\lambda _{m}|c_{j}^{m}|^{2}E_{m}\otimes \theta _{j}, \end{aligned}$$

4.5 $$\begin{aligned} \Phi _{k0}= & {} \rho _{k}\otimes \Lambda ^{*}\rho _{k}=\sum _{m}\lambda _{m}E_{m}\otimes \sum _{n}\lambda _{n}\Lambda ^{*}E_{n}=\sum _{n,m} \sum _{j=1}^{n}\lambda _{n}\lambda _{m}|c_{j}^{m}|^{2}E_{m}\otimes \theta _{j}, \end{aligned}$$

where $\theta _{j}=|Z_{\sigma _{j}}^{(2)}\rangle \langle Z_{\sigma _{j}}^{(2)}|\in {\mathcal {G}}({\mathcal {H}})$, *E* stands for a Schatten decomposition $\{E_{i}\}$ of $\rho _{k}$ and $\lambda _{i}$ be a eigenvalue of $\rho _{k}$.

According to definition 2.4 and lemma 2.5, we can calculate the local quantum mutual entropy as follows:

$$\begin{aligned} Tr\left( {\Phi _{{kE}} \log \Phi _{{kE}} } \right) & = \sum\limits_{{n,m}} {\sum\limits_{{n',m'}} {\left\langle {Z_{{\sigma _{n} }} \otimes Z_{{\sigma _{m} }} ,\Phi _{{kE}} \log \Phi _{{kE}} Z_{{\sigma _{{n'}} }} \otimes Z_{{\sigma _{{m'}} }} } \right\rangle } } \\ & = \sum\limits_{{n,m}} {\sum\limits_{{n',m'}} {\left\langle {Z_{{\sigma _{n} }} \otimes Z_{{\sigma _{m} }} ,\sum\limits_{{p = 0}}^{N} {\sum\limits_{{j = 0}}^{p} {\lambda _{p} } } \mathrm{\mid }c_{j}^{p} \mathrm{\mid }^{2} E_{p} \otimes \theta _{j} \log \sum\limits_{{i = 0}}^{N} {\sum\limits_{{j = 0}}^{i} {\lambda _{i} } |c_{j}^{i} |^{2} |E_{i} \otimes \theta _{j} Z_{{\sigma _{{n'}} }} \otimes Z_{{\sigma _{{m'}} }} } } \right\rangle } } \\ & = \sum\limits_{{n,m}} {\sum\limits_{{n',m'}} {\sum\limits_{{j,p}} {\lambda _{p} } } } |c_{j}^{p} |^{2} \left\langle {Z_{{\sigma _{n} }} \otimes Z_{{\sigma _{m} }} ,E_{p} \otimes \theta _{j} \log \sum\limits_{{i = 0}}^{N} {\sum\limits_{{j = 0}}^{i} {\lambda _{i} } } |c_{j}^{i} |^{2} |E_{i} \otimes \theta _{j} Z_{{\sigma _{{n'}} }} \otimes Z_{{\sigma _{{m'}} }} } \right\rangle \\ & = \sum\limits_{{n,m}} {\sum\limits_{{n',m'}} {\sum\limits_{{j,p}} {\lambda _{p} } } } |c_{j}^{p} |^{2} \left\langle {Z_{{\sigma _{n} }} ,E_{p} Z_{{\sigma _{{n'}} }} \rangle \langle Z_{{\sigma _{m} }} ,\theta _{j} Z_{{\sigma _{{m'}} }} } \right\rangle \log \sum\limits_{{i = 0}}^{N} {\sum\limits_{{j = 0}}^{i} {\lambda _{i} } } |c_{j}^{i} |^{2} \times \left\langle {Z_{{\sigma _{{n'}} }} ,E_{i} Z_{{\sigma _{n} }} } \right\rangle \left\langle {Z_{{\sigma _{m} }} ,\theta _{j} Z_{{\sigma _{{m'}} }} } \right\rangle \\ & = \sum\limits_{{n,m,j}} {\lambda _{n} } |c_{j}^{n} |^{2} E_{n} \otimes \theta _{j} \log \sum\limits_{{n,m,j}} {\lambda _{n} } |c_{j}^{n} |^{2} E_{n} \otimes \theta _{j} ~~\quad \left( {i = p = n = n',j = m = m'} \right) \\ & = \sum\limits_{{n,m,j}} {\lambda _{n} } |c_{j}^{n} |^{2} E_{n} \otimes |Z_{{\sigma _{j} }}^{{(2)}} \rangle \left\langle {Z_{{\sigma _{j} }}^{{(2)}} |\log \lambda _{n} |c_{j}^{p} |^{2} E_{n} \otimes |Z_{{\sigma _{j} }}^{{(2)}} } \right\rangle \langle Z_{{\sigma _{j} }}^{{(2)}} | \\ & = \sum\limits_{{n,j}} {\lambda _{n} } |c_{j}^{n} |^{2} |Z_{{\sigma _{n} }}^{{(1)}} \rangle \left\langle {Z_{{\sigma _{n} }}^{{(1)}} | \otimes |Z_{{\sigma _{j} }}^{{(2)}} } \right\rangle \left\langle {Z_{{\sigma _{j} }}^{{(2)}} |\log \sum\limits_{{n,j}} {\lambda _{n} } |c_{j}^{n} |^{2} |Z_{{\sigma _{n} }}^{{(1)}} \rangle \langle Z_{{\sigma _{n} }}^{{(1)}} | \otimes |Z_{{\sigma _{j} }}^{{(2)}} } \right\rangle \langle Z_{{\sigma _{j} }}^{{(2)}} | \\ & = \sum\limits_{{j,n}} {\lambda _{n} } |c_{j}^{n} |^{2} log\sum\limits_{n} {\lambda _{n} } |c_{j}^{n} |^{2} . \\ \end{aligned}$$

In the same way

$$\begin{aligned} Tr(\Phi _{kE}log\Phi _{k0})=\sum _{j,n}\lambda _{n}|c_{j}^{n}|^{2}log|c_{j}^{n}|^{2}, \end{aligned}$$

Finally, applying the relation $S(\Phi _{kE}\Vert \Phi _{k0})=Tr(\Phi _{kE}\log \Phi _{kE})-Tr(\Phi _{kE}log\Phi _{k0})=\Sigma _{i}\nu _{i} S(\Lambda ^{*}E_{i}\Vert \Lambda ^{*}\rho _{k})$ and lemma 2.5, we obtain the local quantum mutual entropy

$$\begin{aligned} I(\rho _{k};\Lambda ^{*})=\sum _{j,n}\lambda _{n}|c_{j}^{n}|^{2}log(\sum _{n}\lambda _{n}|c_{j}^{n}|^{2}/|c_{j}^{n}|^{2}). \end{aligned}$$

## Summary

In this paper, we give the mathematical structure of the communication channel when the noise is local quantum Bernoulli noises, and prove that the channel is completely positivity and trace-preserving property map. Local quantum mutual entropy represents the maximum amount of correct information from the input system to the output system. However, the mutual entropy is a measure for not only information transmission but also description of state change, since this mutual entropy can be applied to several aspects of quantum dynamics, it can also be applied to quantum computers or some topics in computers to look at the ability to transmit information.

Applying the Schrodinger equation, we calculate that the local quantum mutual entropy is $\sum _{j,n}\lambda _{n}|c_{j}^{n}|^{2}log(\sum _{n}\lambda _{n}|c_{j}^{n}|^{2}/|c_{j}^{n}|^{2})$, that is, the maximum amount of correct information transmitted when the noise is local quantum Bernoulli noise. Applications of the mutual entropy can be found in various fields^8,10–16^. The mathematical discussion of a channel was given in^8,17,18^, and details of the noisy channel were given in^16^.

## Data Availability

Data sharing is not applicable to this article as no data sets were generated or analyzed during the current study.
